# Integration of Myeloblastosis Associated Virus proviral sequences occurs in the vicinity of genes encoding signaling proteins and regulators of cell proliferation

**DOI:** 10.1186/1478-811X-4-1

**Published:** 2006-01-10

**Authors:** Chang Long Li, Philippe Coullin, Alain Bernheim, Véronique Joliot, Charles Auffray, Rima Zoroob, Bernard Perbal

**Affiliations:** 1 Laboratoire d'Oncologie Virale et Moléculaire, Case 7048, UFR de Biochimie, 2 place Jussieu, Université Paris 7 D. Diderot, 75005 Paris, France; 2 Laboratoire de Cytogénétique and CNRS UMR 8125, Institut Gustave Roussy, 94805 Villejuif, France; 3 Unite de Génétique Moléculaire et de Biologie du Développement (CNRS UPR 420), 94801 Villejuif, France; 4 Endocrinologie et génétique du développement et de la reproduction INSERM U 782 92140 Clamart (France); 5 Cellular regulations and oncogenesis-UMR 146 CNRS/Institut Curie; 6 Genexpress, Functional Genomics and Systems Biology for Health, CNRS UMR 7091-7, 94801 Villejuif Cedex, France; 7 UPR 1983, CNRS, 7 rue Guy Moquet, 94801, Villejuif Cedex, France

## Abstract

**Aims:**

Myeloblastosis Associated Virus type 1 (N) [MAV 1(N)] induces specifically nephroblastomas in 8–10 weeks when injected to newborn chicken. The MAV-induced nephroblastomas constitute a unique animal model of the pediatric Wilms' tumor. We have made use of three independent nephroblastomas that represent increasing tumor grades, to identify the host DNA regions in which MAV proviral sequences were integrated. METHODS Cellular sequences localized next to MAV-integration sites in the tumor DNAs were used to screen a Bacterial Artificial Chromosomes (BACs) library and isolate BACs containing about 150 kilobases of normal DNA corresponding to MAV integration regions (MIRs). These BACs were mapped on the chicken chromosomes by Fluorescent In Situ Hybridization (FISH) and used for molecular studies.

**Results:**

The different MAV integration sites that were conserved after tumor cell selection identify genes involved in the control of cell signaling and proliferation. Syntenic fragments in human DNA contain genes whose products have been involved in normal and pathological kidney development, and several oncogenes responsible for tumorigenesis in human.

**Conclusion:**

The identification of putative target genes for MAV provides important clues for the understanding of the MAV pathogenic potential. These studies identified ADAMTS1 as a gene upregulated in MAV-induced nephroblastoma and established that ccn3/nov is not a preferential site of integration for MAV as previously thought. The present results support our hypothesis that the highly efficient and specific MAV-induced tumorigenesis results from the alteration of multiple target genes in differentiating blastemal cells, some of which are required for the progression to highly aggressive stages. This study reinforces our previous conclusions that the MAV-induced nephroblastoma constitutes an excellent model in which to characterize new potential oncogenes and tumor suppressors involved in the establishment and maintenance of tumors.

## Introduction

Chicken nephroblastomas induced by MAV1-N represent a unique animal model of the Wilms tumor, a kidney cancer occurring in young children at a frequency of about 1:6000 births. Early cytogenetic studies have identified multiple chromosomal alterations in Wilms tumors, raising the possibility that several steps in the differentiation pathway of blastemal cells could represent potential targets for tumorigenic events [1].

In an attempt to characterize genes that are altered at various stages of tumor development, we have taken advantage of the histological similarities between Wilms tumor and MAV-1(N) induced nephroblastoma.

MAV is a replication competent retrovirus which can induce nephroblastomas, osteopetrosis and lymphoid leucosis when injected in chicken [2]. Molecular cloning of the MAV1(N) proviral genome permitted us to isolate a pure viral strain inducing specifically nephroblastomas when injected, either intravenously in ovo on embryonic day 18, or intraperitoneally in day-old chickens [3]. The characterization of MAV sequences contained in avian nephroblastomas established that these tumors were polyclonal and that in tumor DNA, MAV was inserted at a limited number of sites, suggesting that either the integration of MAV at other sites was not associated with nephroblastoma induction, or that the selection pressure occuring naturally during tumor development had counterselected cells carrying MAV proviral genomes at other sites [1,4].

The analysis of lambda librairies obtained from these tumors reinforced the idea that in the tumor DNA, the MAV proviral sequences were integrated at a few distinct cellular sites. The MAV genomes present in well-developed tumors were all heavily rearranged whereas in diffuse tumors of smaller size showing a less advanced tumor phenotype, the MAV genomes were full length in size and functional [5].

Use of junction fragments containing viral U3 and adjacent cellular sequences, permitted to establish that in one of the most developed tumor one of the proviral genome was integrated within a gene that is known as ccn3 and that we originally designated "nov" for « nephroblastoma overexpressed » [5].

CCN3 is one of the three founders of the CCN family of proteins which presently consists of six different members. Its expression is associated with cell quiescence [6,7]. In normal conditions, the expression of ccn3 undergoes spatiotemporal regulation in several different tissues originating from the three germ layers, with major sites of expression being adrenal, nervous system, cartilage and bone, muscle, and kidney [7-14]. The production of CCN3 protein can be increased or decreased upon carcinogenesis [7,12,15-20]. In Wilms' tumors, the expression of ccn3 was a marker of differentiation [12] whereas in Ewings' tumors, the expression of ccn3 was associated with a higher risk of developing metastasis [17]. In all cases, the full length CCN3 protein shows antiproliferative activity.

Albeit its expression was elevated in all avian tumors, the ccn3 gene was found to be disrupted in only one case, suggesting that either an unknown viral product, or MAV LTR enhancer was responsible for increased ccn3 expression. Indeed, it is well known that LTR enhancer sequences can activate transcription of genes localized several tens of kilobases away. However, the limited size (20 kb) of the insert DNA that was contained in the lambda recombinants, did not permit to establish whether MAV LTR sequences were present in the vicinity (at a genome scale) of the ccn3 gene in the DNA of all tumor cells.

Since we had isolated and studied tumors representing three increasing developmental stages, we took advantage of this material to ask whether the progression from an initial diffuse tumor to a well developed tumor, was accompanied by the selection of cells carrying particular MAV integration sites. To tackle this problem, we have used the BAC (bacterial artificial chromosome) and FISH (fluorescent in situ hybridization) strategies.

The results we report here confirm that a limited number of MAV-integration sites are detected in the DNA of MAV-induced nephroblastomas, with an over representation of integration sites on chromosome 2. In well-developed tumors, MAV-integration sites are localized in the vicinity of genes encoding proteins involved in matrix remodeling, angiogenesis, and signaling. Our results also indicated that ccn3 is not a common integration site in these tumors.

## Materials and methods

### Labeling of the BAC DNA fragments

Prior to labelling, the BAC DNA fragments were amplified by PCR using the Expand high fidelity PCR system from BOEHRINGER MANNHEIM. One hundred nanograms of insert was mixed with U and R primers (0.3 μM/L), 8 μl dNTP(10 mM), mix II buffer (10 μl), TaqE (3 U) and water to 50 μl. Amplification was performed for 30 cycles of 94°C for 30 seconds, 50°C for 1 min, 72°C for 1 min. The size of the amplified fragments were checked by electrophoresis in 1% agarose gels prior to purification with the QIAquick PCR Purification Kit (Qiagen). DNA fragments (50 ng of each) were labeled with the Amersham multiprime DNA labelling system (Amersham Pharmacia Biotech RPN161Z LIFE SCIENCE) in the conditions recommended by the supplier and purified by filtration through Sephadex G50 to remove nucleotides that were not incorporated.

### Screening of the BAC library

Duplicate filters on which BAC DNA preparations had been transfered were incubated with labelled probes as described above. Colonies containing positive BACs were picked and grown at 37°C overnight into 4 μ of LB medium containing 10 μg/ml chloramphenicol. The DNA contained in pelleted cells was extracted as described above and resuspended in 40 μl of TE containing RNaseA. The DNA content of each BAC was analyzed by both Dot blotting and Southern blotting of HindIII-digested DNA.

### Ccn3 probe

The pC1K clone [5] was used as a source of chicken ccn3. For preparation of the ccn3 probe, the 2.0 kb Kpn1fragment was purified by electroelution as described [21]

### Isolation of polyadenylated RNA from normal kidneys and nephroblastomas

Frozen tissues were homogenized with a polytron and 0.5 g of powder was resuspended in 9 ml guanidine thiocyanate buffer for purification of total RNA as previously described [21]. Final RNA pellets were resuspended in 400 μl sterile distilled water and the concentration of each sample was determined by densitometry. To isolate polyadenylated RNA species, each sample (1 mg total RNA in 500 μl water) was mixed with 55 μl Oligitex Suspension (Qiagen) and incubated for 3 min at 70 min in a water bath. After 10 min at room temperature the Oligotex:mRNA complex was pelleted by 2 min centrifugation at 14000–18000 g and the supernatant carefully removed. The pellet was further treated as recommended by the supplier and the polyadenylated RNA fraction was collected in a final volume of 50 μl.

### Labelling of polyadenylated RNA preparations

To prepare labelled RNA probes, 500 ng of each polyA-RNA preparation were mixed with 500 ng oligo dT, incubated for 10 min at 70°C and chilled on ice for 5 min. Samples were then mixed with 5 μl of 10 × PCR buffer, 5 μl of MgCl2 25 mM, 5 μl DTT 0.1 M, 2.5 μl mixture of dTTP, dATP, dGTP(10 mM each), 2.5 μl of ddTTP(1 mM), 5 μl of ^32^P-dCTP, and incubated for 5 min at 25°C. After addition of 1 μl of reverse transcriptase (Invitrogen) (200 U/ μl), the mix was incubated for 10 more min at 25°C and for 50 min at 42°C. The reaction was stoped by incubation at 70°C for 15 min. Each labelled preparation was purified by chromatography through a column of Sephadex G50.

### Hybridization of BAC DNA filters

The blots were rinsed with 6 × SSC, and prehybridized at 68°C for 18 hours. After hybridization with labeled probe in the presence of COT I DNA the blots were washed with 2 × SSC, 0.1%SDS at 56°C for 1 hour and with: 0.1 × SSC, 0.1%SDS at 65°C for 1 more hour. Autoradiography of the dried blot was performed at -80°C.

### Purification of BAC DNA fragments

To recover the DNA fragments containing sequences that encode differentially expressed RNAs, 4 μg of BAC DNA were digested with HindIII, and run in 1% low melting agarose gel. The fragments of interest were eluted by incubation at 65°C for 10 min prior to addition of 1 ml Wizard Plus resin from Promega and filtration through a minicolumn connected to vacuum manifold. The column was rinsed with washing buffer and the DNA fragments eluted with 50 μl of TE buffer.

### Ligation and transformation

HindIII digested purified DNA fragment (50–100 ng) were ligated to 50 ng of dephosphorylated HindIII-digested pUC18 vector in the presence of 2.5 μl T4 ligase (Appligene) at 14°C for 18 hours. For transformation, 2 μl samples of the ligation mixture were mixed with 200 μl of DH5α competent cell. Electroporation was performed at 2.45 Kv, 25 μF, 400 Ohm. After addition of 1 ml of cold LB medium, bacteria were spread onto LB plate containing 100 μg/ml ampicillin and incubated at 37°C.

### Screening of chicken cDNA libraries

A library of chicken spleen cDNA was spread on LB agar plates containing tetracycline(7.5 μg/ml) and ampiciline(12.5 μg/ml) and incubated at 37°C, overnight. The colonies were transferred to Qbiogene Neutral Membranes and replicated to LB agar plates containing tetracycline and ampiciline. The membranes were incubated successively in 0.5 N NaOH for 10 min, 0.2 N NaOH and 1.5 M NaCl for 10 min, 0.2 M TrisHCl and 2 mM EDTA for 20 seconds and 2 × SSC for 20 seconds. The filters were baked at 80°C for 2 hours prior to use for hybridization with appropriate probes.

### Sequencing of positive cDNA clones

Sequencing of the cDNAs was performed with T7 universal primer and CDM8 (TAAGGTTCCTTCACAAA) primer.

### Northern blot hybridization

Samples of total RNA (20 μg in 9.3 μl water) were incubated with, 20 μl deionized formamide, 6.7 μl formaldehyde, 4 μl 10 × MOPS, incubated for 5 min at 68°C, chilled on ice before loading. Formaldehyde-MOPS gels were run at 100 volts for loading and 50 volts overnight in 1 × MOPS buffer. The gel were then rinsed with DEPC-treated 20 × SSC containing 2 mercaptoethanol, transferred to Appligene positive Membranes and treated for hybridization.

Prehybridization was performed in 50% formamide, 5 × SSC, 3 x Denhardt, 0.5% SDS, 0.05 M NaH_2_PO4, salmon sperm DNA 100 μg/ml, at 42°C for 3 hours. Hybridization was performed in the same solution containing probes(1–3,000,000 cpm/ml). Washing of the membrane was performed in 2 × SSC, 0.2% SDS.

### Cultures and chromosome preparation

Chicken (Gallus gallus) cells were obtained from 9-days-old embryos using trypsin. The cultures were incubated at 37°C or 41°C in MEM medium supplemented with 20% foetal calf serum, glutamine and penicillin/streptavidin. For cytogenetic preparations, the cells were collected using trypsin and treated with an hypotonic solution: KCL (2.8 g/l), foetal calf serum 1/12 and they were fixed with 3:1 methanol/glacial acetic acid. The same procedure was used for the other galliformes tested namely: the Common Quail (*Coturnix coturnix*), the Red-legged Partridge (*Alectoris rufa*), the Grey Peacock-Pheasant (*Polyplectron bicalcaratum*), the Cheer Pheasant (*Catreus wallichi*), the Common Pheasant, (*Phasianus colchicus*), the grey partidge (*Perdix perdix*), the turkey (*Meleagris gallopavos*) and the guinea-fowl (*Numida melagris*).

### Fluorescent in situ hybridization (FISH)

The purified DNA inserts were labelled by nick translation with biotin or digoxigenin 16 dUTP (Appligene OncorR nick-translation kit, Illkirch Graffenstaden, France) according to manufacturer's instructions.

Chromosomes slides were incubated at 70°C for 2 min in 70% formamide, 2 × SSC (pH 7.2) and dehydrated in an ethanol series at 4°C. 2 μl of a 1/5 dilution of labelled probe was mixed with 6 μl of human Cot1 DNA(from a 1 mg/ml solution ; GibcoBRLR) and 32 μl of hybrisol VI (Oncor R) denatured 5 min at 80°C and incubated 30 min at 37°C before deposition on the slide. The slides were then incubated overnight at 37°C in a humidified chamber and washed three times 5 min at 42°C in 50% formamide, and in 2 × SSC at 42°C. After being rinsed in 4 × SSC at room temperature, the slides were incubated for 30 min at 37°C in blocking solution (Roche Diagnostics Meylan France). The digoxigenin was detected using an anti-digoxigenin-rhodamin-labelled antibody (Appligene OncorR) and the biotin with FITC labelled avidine (Appligene OncorR).

The slides were washed with 4 × SSC for 10 min in a shaker. After draining the excess liquid, DAPI was used as counterstain and Vectashield R (Vector laboratories Inc. Burlingame, CA 94010, USA) as antifading solution. Pictures were acquired on a Zeiss epifluorescence microscope using a tri-CCD camera and Vysis computer software (Smart capture 2). Chromosome assignments were made using reverse DAPI by reference to the GTG banded ideograms of chicken proposed by Ladjali-Mohammed et al [22].

The cytogenetic localization of the various BAC DNAs was performed by two independent FISH runs. For each experiment 50, and 25 cells were analyzed respectively. Except for rare cases, four chromatids per cell showed a specific signal and the percentage of triploid and tetraploid cells present in the embryonic chicken fibroblast culture was taken into account.

### Human-chicken genomic comparisons

Syntenic conserved chromosome segments between human and chicken were determined from Schmid et al [23]. Human cancer genes localized in the syntenic areas were selected from the "Atlas of Genetics and Cytogenetics in Oncology and Hematology". . Their presence in the presumed chicken chromosome areas was investigated by data processing using different web sites (NCBI:, EMBL EBI : , infobiogène :  for human and NCBI, Wageningen university :  for chicken) and by calculation from the physical to the cytogenetical localisation using the relative position in the sequence and the " consensus mid-points " of the markers reported in Schmid et al [23].

## Results

### Isolation of BACs clones harbouring genomic DNA fragments flanking the viral/cellular junctions of MAV1-related proviruses

**Figure 1:**
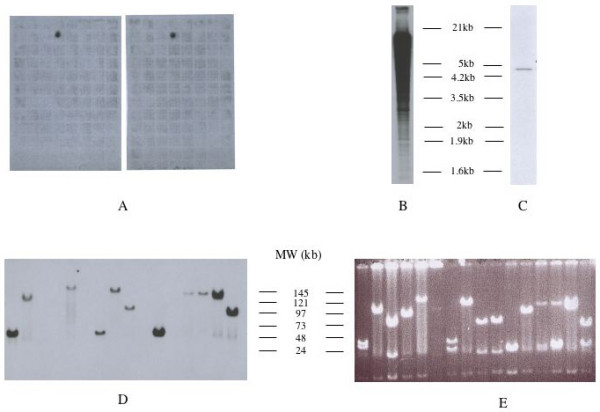
Isolation and characterization of BACS. Panel A shows typical results obtained with a BAC containing a MAV-integration site contained in one of the probes dereived from avian nephroblastoma. Filters of BAC DNA were duplicated. To check the specificity of the probes used, two micrograms of genomic chicken DNA were digested with 40 units of HindIII restriction endonuclease at 37°C for 18 hours and run in a 1% agarose gel at 2 volts/cm for 20 hours. The separated DNA fragments were denatured by incubation in 0.5 N NaOH for 45 min, neutralized in 0.5 M Tris HCl, 1.5 M NaCl. Transfer onto Appligene Positive Membrane was performed in 20 × SSC for 18 hours and the membrane was baked for 2 hours at 80°C prior to use for hybridization with labeled cloned cellular fragments. All cellular probes cloned from nephroblastoma DNA libraries detected a single fragment in HinIII digested normal DNA (see panel C for typical result) except for P38 which contained chicken repetitive sequences (panel B). Panels D and E: DNA preparations from positive Bacs were digested with NotI (panel 1 E shows ethidium bromide staining of the gel) and transfered onto nitrocellulose prior to hybridization with the probes used for their isolation. A single NotI fragment is detected by the probes in the BAC dans (panel 1D).

From the libraries of lambda recombinant DNA prepared with tumors 501D, 501 and 725 [5] we derived a total of 23 DNA fragments containing genomic sequences flanking the junction fragments that were previously identified in these three MAV-induced tumors. These fragments are representative of MAV Integration Regions (MIRs) at the chromosomic scale. Their size ranged from 800 bp to 4. 7 kb. Each of them was checked by Southern blot analysis on chicken genomic DNA for the presence of repetitive sequences (figure 1B,C). Only one of them was found to contain repetitive sequences (figure 1B) and was discarded. The 22 remaining clones were used as probes for screening a chicken BAC (Bacterial artificial chromosome) library which contain 60.000 clones arrayed in 96-well plates and with an average insert sizes of 120–150 kb (figure 1A). The probes were pooled by groups of three or four prior to hybridization. A total of 78 positive clones were selected for further studies, the DNA of which was digested with Not I and hybridized with the different probes separately to verify that they indeed contained MIRs (figure 1D,E).

**Figure 2:**
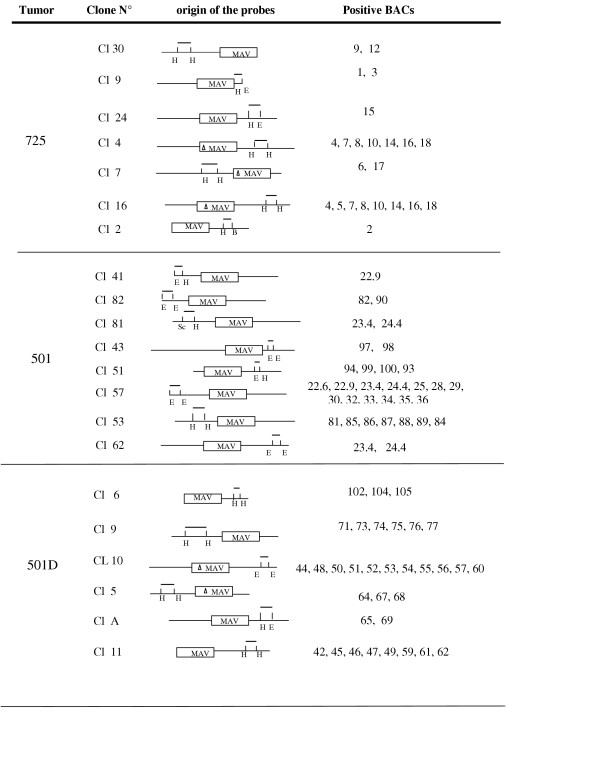
Listing of positive BACs. All positive BACs are listed with the origin of the probes used for their isolation. The three groups corrrespond to tumors representing increasing developmental stages.

Three different groups of BACs were isolated (figure 2) with the probes originating from the three nephroblastomas. Each group was tumor-specific. None of the probes from one given tumor, hybridized with BACs corresponding to another tumor (data not shown). Furthermore, when all BACs from each group were radiolabeled and used as probes on BACs from the two other groups, no overlaping sequence could be found (data not shown). These results indicated that no DNA fragment was common to the collection of 78 BACs. Interestingly probes 4 and 16 hybridized with several different BACs (4,7,8,10, 14,16) in the same group, and probes 81 and 62 hybridized with two different BACs (23.4 and 24.4) that belong to another group. These results suggested that two integrations sites of MAV were localized in the same DNA fragments in tumor 725 and in tumor 501. However, they correspond to different MIRs in the chicken genome.

In order to localize the various MIRs at a chromosomic scale, we have performed FISH experiments using the various BACs as probes.

### Chromosome localization of BACs

**Figure 3:**
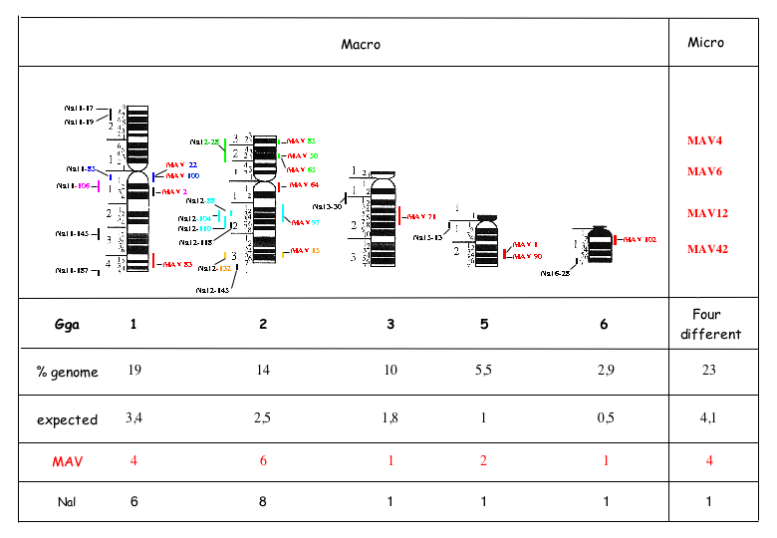
Distribution of positive BACs on chicken chromosomes.

Eighteen BACs, containing sequences detected with the different probes could be assigned on the chicken chromosomes (figure 3). Although the distribution of MIRs on macro and micro-chromosomes corresponded to the expected theoretical value the number of MIRs mapped on chromosome Gga2 was twice as larger as the number that would be expected from a random distribution.

**Figure 4:**
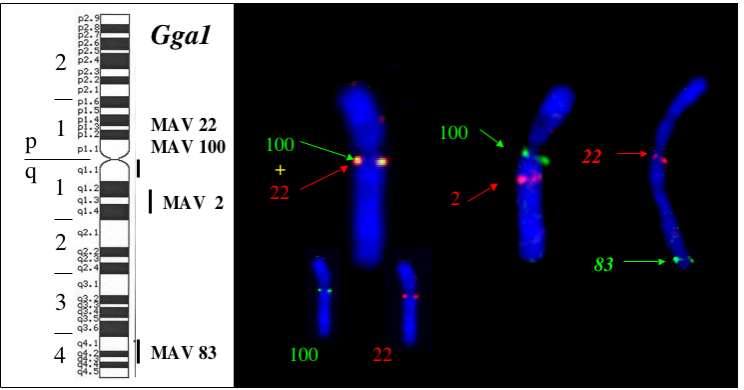
Mapping of BAC sequences on chicken chromosome 1.

**Figure 5:**
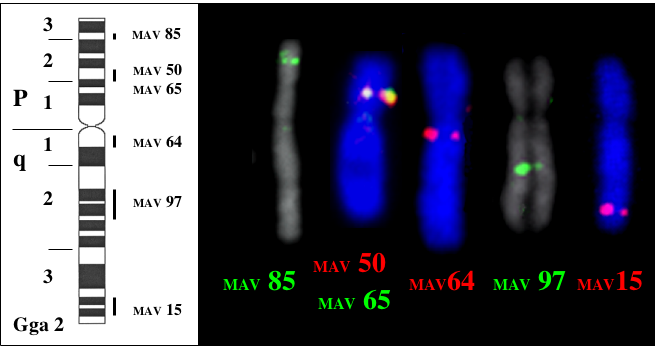
Mapping of BAC sequences on chicken chromosome 2.

**Figure 6:**
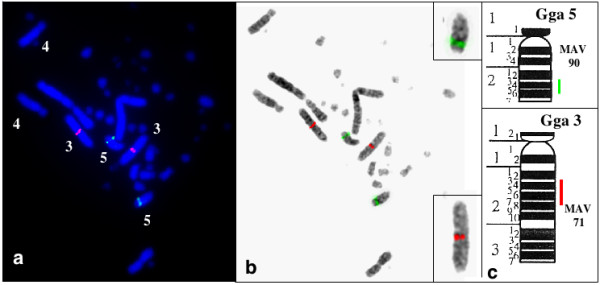
Mapping of BAC sequences on chicken chromosome 3 and 5.

Four BACS (2, 22, 83 and 100) were mapped on chromosome Gga1 (figure 4). Six BACs (15, 50, 64, 65, 85 and 97) mapped on Gga 2 (figure 5). BAC 15 contains the ccn3 locus which was previously assigned to Gga2 [28]. Two BACs (1 and 90) mapped on Gga5 and BACs 71 and 102 were maped on Gga 3 and 6 respectively (figure 6)

The precise identification of the micro-chromosomes that gave a positive signal was not performed but co-hybridization experiments established that the four positive BACs corresponded to loci which were localized on 4 different microchromosomes (data not shown).

**Figure 7:**
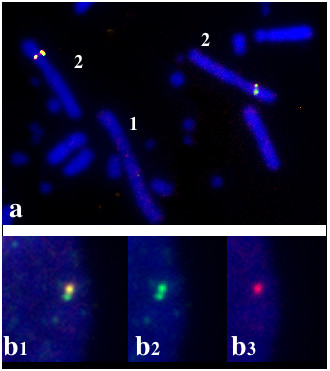
Co-localisation and assymetrical duplication of areas identified by BAC 50 (red) and 65 (green).

It is worth noting that the DNAs from 2 Bacs (22 and 100) which were isolated with three different cellular probes from tumor 501 (41, 57 and 51 respectively) co-localized on Gga 1q11 (figure 4) and could not be separated on interphasic nuclei. From these results, one could estimate that the distance separating the sequences contained in BACs 22 and 100 is smaller than a thousand kilobases. Similarly DNA sequences from BACs 50 and 65 which were detected by probes 10 and 18 co-localised on Gga 2q21 (figure 7). In that case, the analysis of asynchronus replicative figures allowed us to establish that the corresponding loci were distinct and probably contained in a DNA segment of a few hundred kilobases. Most interestingly, two BACs (1 and 90) isolated with probes from two different tumors (725 and 501) were assigned to Gga 5q23-25.

The strong signals obtained after hybridization of the chicken BACs with other galliform chromosomes suggested that the sequences of the MAV targets are relatively well conserved throughout the galliform order. The chromosome assigments were identical, with respect to karyotypes evolution among these birds. As an example, markers of Gga2 are scattered on two chromosomes in Castreus wallishii (data not shown).

**Figure 8:**
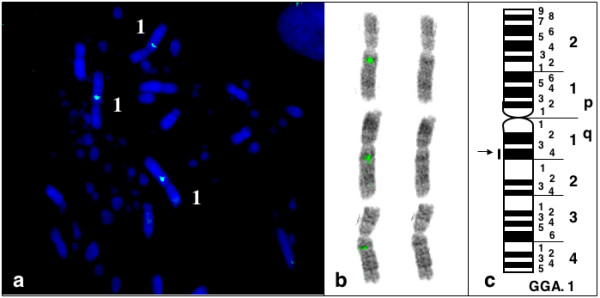
Maping of BAC2 on Gga1q14 (triploid metaphase).

We have localized BAC2 which carries the chicken AdamTS1 gene on GGA1q12-q14 region, most probably in q14 (figure 8). The physical distance (fractional length measurement or Flpter) for the Adam TS1 gene is 542. To date, a series of 6 genes have been assigned on chicken chromosome GGA1q12-q14: IFNAR2, IL10R, IFNAR1, GART (q12-q13); SOD1; CRYAA q14 of which the human heterologs lie on chromosome segment Hsa21q22.1-q22.3. Excepted for CRYAA which segregated on Chromosome Mmu17, all these markers were found together in the mouse on chromosome Mmu16 [23]. Human and mouse AdamTS1 were respectively assigned on HSA21q21.3 [32] and MMU16.

The assignment of ccn3 on Gga 2q34-36 was already reported [28]. To date, a series of nine genes have been assigned on chicken chromosome 2q: PRKDC (q24-25), PENK, MOS, LYN (q26), CALB1 (q26), CA2, TRHR, MYC and HSF1 of which the human homologs lie on chromosome segment 8q11-q24.1[23]. The localization of ccn3 on human 8q24 and chicken chromosome 2q34-36 reinforced the chromosomal homology between the two species and suggested that the syntenic segment between the two species could be extended up to avian 2q3. Furthermore, the mouse ccn3 gene maps to chromosome 15 [33] in a region of conserved synteny with man including TRHR, MYC and HSF1 [23].

The order of genes on the chicken map is still subject to changes. Furthermore, based on detailed analyses on other chicken chromosomes many rearrangements are known to occurr within syntenic regions. However, with the restriction regarding the order of the genes, the present findings suggest that ccn3 and AdamTS1 belong to syntenic groups well conserved between chicken, mouse and man. These genes also constitute another example where the synteny is better conserved between chicken and man than between man and mouse.

### ADAMTS1 sequences are overexpressed in MAV-induced nephroblastomas

The detection of ADAMTS1 locus as a MAV integration site caught our attention because the ADAMTS1 protein belongs to a family of proteases involved in angiogeneis and tissue remodeling that are both required for tumor progression [32]

In order to establish whether the BACs of interest indeed contained genes differentially expressed in tumor samples, polyadenylated RNA species purified from normal kidneys and nephroblastomas, were used to probe digested BAC-DNAs.

**Figure 9:**
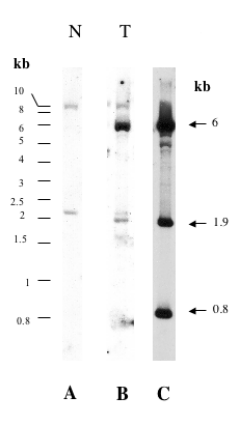
Detection of CCN3 sequences in normal and tumor DNA. RNA species purified from normal kidney cells (N) and tumor cells (T) were labeled (see materials and methods) and used to probe BAC15 DNA that harbors the ccn3 gene. The DNA fragments detected with the RNA species expressed in tumor cells (panel B) confirm that ccn3 is overexpressed in the tumor context. As a control, the DNA fragments from BAC15 were hybridized with radiolabeled chicken ccn3 cDNA. The DNAfragments which are detected correspond to exons encoding the ccn3 RNA species that are highly expressed in the tumor context (panel C).

As a control we first checked that hybridization performed with these labeled RNA species permitted detection of the DNA fragments of BAC15 which contained the CCN3 gene [28] known to be overexpressed in all MAV-induced nephroblastomas. Indeed, the size of the DNA fragments whose detection was highly increased after hybridization with tumor-derived RNA species corresponded to the size of the genomic CCN3 Hind III fragments that was previously drawn from the characterization of the ccn3 chicken locus (figure 9 and ref1).

**Figure 10:**
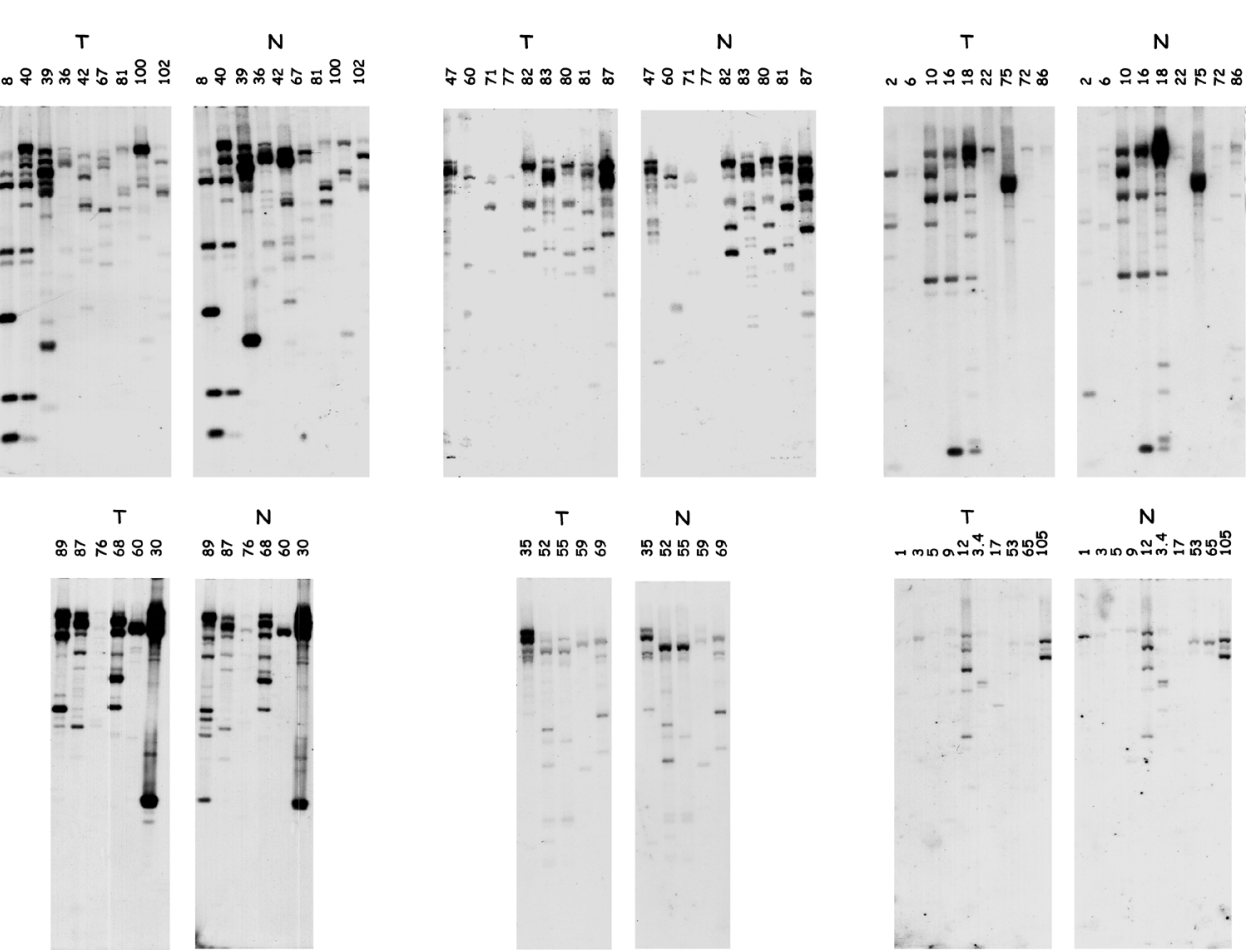
Differential expression of genes contained in positive BACs. The digested BACs DNAs were hybridized with labeled RNA species isolated from either normal kidney cells (N) or tumor cells (T). Comparison of the hybridization patterns allowed to identify DNA fragments containing genes whose expression is either enhanced or abolished in tumors.

Typical results obtained with a series different BACs are shown in figure 10. Various BACs contained DNA sequences encoding RNA species that were either decreased or increased in tumors, or both.

**Figure 11:**
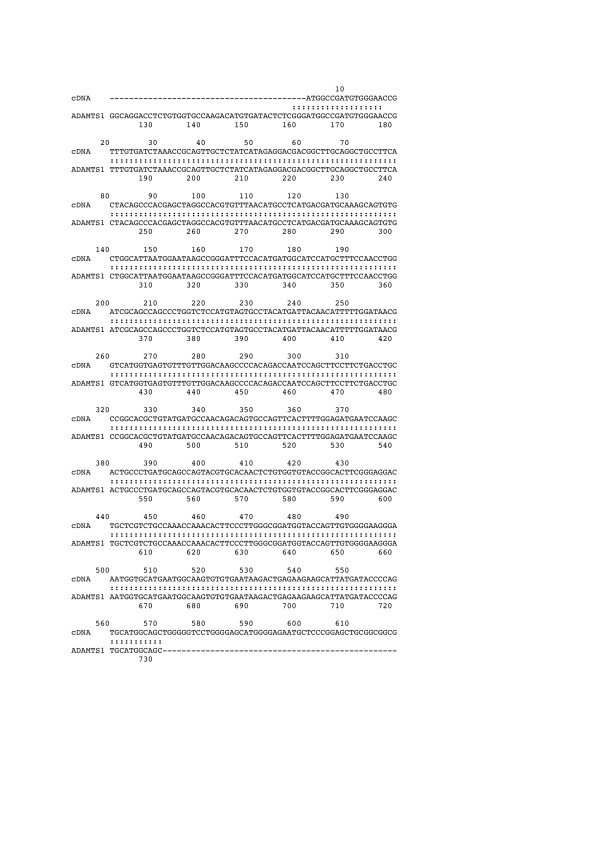
ADAM TS1 is contained in the 6.2 kb DNA fragment from BAC2. The nucleotide sequence of the mRNA species encoded by the 6.2 kb DNA fragment identified by differential hybridization of BAC2 with tumor and normal RNA species is aligned with the human ADAM TS1 sequence.

**Figure 12:**
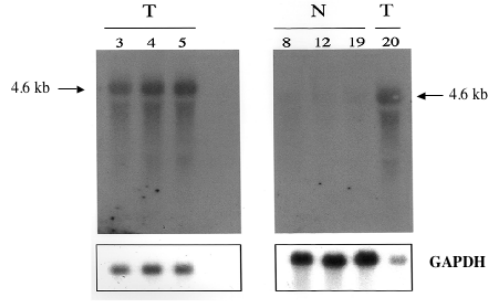
Detection of ADAMTS1 expression in normal kidney and nephroblastoma tissues. RNA samples 3,4, 5 and 20 were prepared from MAV1-induced nephroblastomas collected 18 weeks after injection of MAV1 and RNA samples 8,12, and 12 were prepared from normal kidney of 18 week old chicken. Electrophoresis and Northern blotting were performed as described in the text. The resulting blot was hybridized under stringent conditions with radiolabeled cDNA corresponding to the chicken ADAMTS1 sequence. Samples of glyceraldehyde 3 phosphate dehydrogenase RNA were used as quantitation standard.

As a first step in our identification of MIRs, we focused on BAC2 because it contained sequences mapping in the vicinity of ADAMTS1, and it provided a simple differential hybridization pattern. Two DNA fragments (6.2 kb and 3.2 kb) encoded abundant RNA species in the tumor samples, whereas a low molecular weight fragment encoded sequences that were slightly reduced in tumors. Only the 6.2 kb and 3.2 kb fragments could be subcloned. When used as probes on chicken DNA, the 3.2 kb fragment was found to contain repetitive sequences and could not be used for further studies. The 6.2 kb fragment of BAC2 could be used as a probe to check that it was indeed strongly detected by RNA from the tumor samples (data not shown). To identify sequences that were expressed from this 6.2 kb DNA fragment, the cloned insert was used as a probe to screen a chicken spleen cDNA library. Sequencing of two positive cDNA clones indicated that they were sharing 100% identity with part of the human ADAMTS1 coding sequence (figure 11). These results indicated that the DNA locus containing the chicken ADAMTS1 gene, was a MAV integration site and suggested that disregulation of ADAMTS1 might be involved in the development of MAV-induced nephroblastomas. Northern blotting of RNA species isolated from normal kidneys and four different MAV-induced nephroblastomas indeed established that ADAMTS1 was overexpressed in the 4 MAV-induced nephroblastomas as compared to normal tissue (figure 12).

## Discussion

The studies that we have performed during the past decade have allowed us to identify viral sequences responsible for the very high efficiency and restricted pathogenic potentential of MAV1-(N) [29-31]. We also established that MAV-induced nephroblastomas were polyclonal tumors [4] that constituted a unique model of the pediatric Wilms tumor [1].

The analysis of genomic libraries prepared from MAV1-induced tumors representing three different stages in tumor progression established that the MAV proviral genomes contained in the DNA of the tumor cells were not integrated in common sites. However, the relatively small size of the DNA insert transduced by the recombinant lambda phages, did not permit to exclude the possibility that MAV proviral genomes were inserted in common regions at the chromosome scale.

In order to determine whether MAV-induced rearrangements of the host genome were common to the three chicken nephroblastoma tumours that represented increasing developmental stages [5], we have isolated and characterized 78 BACs containing the normal DNA fragments corresponding to the insertional sites of MAV in the genome of these tumor cells. The molecular analysis of these BACs did not permit us to identify any common integration sites, but one, among the three different tumors.

It is well known that selective pressures likely occured during tumor progression and that the integration sites that we might identify at the late stages could be associated with events that led to tumor establishment. Therefore the lack of a common integration site, at a scale of 150 kb, probably reflected the various developmental stages and phenotypes of the tumors. On the other hand, our results suggested that preferential MAV integration sites might be conserved in the developed tumors, since independent junction fragments corresponding to different proviral genomes cloned from a given tumor, were hybridizing with the same BACs. These results suggested that the distribution of MAV integration sites in the tumors might not represent initial events but rather reflect the complex chromosome rearrangements that occur during tumor progression.

The use of BACs to perform a FISH analysis of the MAV integration sites permitted us to gain a better insight into the distribution of integration sites in the various tumors that we analyzed. In spite of the polyclonal nature of the nephroblastomas, a rather simple profile was obtained. The chicken genome is composed of 34 chromosomes, among which are 9 macrochromosomes and 25 microchromosomes. The MAV integration sites were found to be equally distributed between the micro and macrochromosomes that stained positive. However, these sites were not distributed randomly. Instead, the number of MAV integration site on chromosome 2 was much higher than expected and 3 integration sites were detected on this chromosome by two independent BACs. These finding suggested that during the establishment and progression of nephroblastomas, the maintenance of chromosome 2 alterations were preferentially selected. The results obtained by FISH confirmed that MAV proviral genomes were integrated in a limited number of sites, as previously predicted by junction fragment analysis [4] and pulse field electrophoresis [1].

During the preparation of this manuscript, Pajer et al. reported the use of inverse PCR and LTR-RACE to identify nephroblastoma-associated loci (Nals) in MAV2-induced avian nephroblastomas (Pajer, personal communication and manuscript in press). In order to compare the position of MAV insertion sites identified by FISH and PCR, we have calculated the physical localization of the Nals on each corresponding chromosome and found a fairly good match between the two sets of results (See additional file 1: Compilation of cytogenetic data obtained from FISH analysis). In both studies, MAV integration sites were found to be mainly distributed among chromosomes 1 and 2. Of particular interest was the identification of hot spots for proviral sequences at 1q1, and 2q2. Whether these sites represent preferential MAV integration sites or regions that contain genes required for tumor development remains an open question.

Two different types of information could be drawn from these observations: i) the distribution of MIRS and Nals points to chromosome regions that frequently harbour proviral MAV sequences in tumors; these regions likely contain genes that are important for tumor development; and ii) the reduced number of MAV integration sites that are maintained during tumor progression points to genes that are probably important at later stages, and the comparison of MAV integration sites in early and late tumors might help to distinguish between genes involved in the establishment and in the maintenance of the tumor state.

Based on the relatively well conserved synteny betwen man and chicken it was also possible to predict the nature of potential genes of interest. The use of normal and tumor RNAs as probes to identify BAC fragments that contain genes that are differentially expressed in normal and tumor tissue (figure 10) also provided critical information that could be used as another clue to assign potential genes to MAV insertion loci.

MAV integration sites were identified by FISH analysis (BACs 50, 65, 64, 97, 15) on Chromosome 2. Among the potential genes of interest contained in these areas Plag1 (8q12 in human), and twist (7p21) are target in 6% and 4% of MAV2-induced tumors (Pajer et al. In ress). Both encode transcription factors that are thought to play a role in tumorigenesis, LRCC (Leiomyomatosis and Renal Cell Cancer, 1q42-44 in human) is associated with papilloma renal cancer, while WTSL (Wilms tumor suppressor locus, 7p14-13 in human) is a potential suppressor gene whose alteration appears to be involved in normal kidney development and nephroblastoma. The CCN3/NOV gene (8q24-1 in human) which is a target for both MAV1 and MAV2-induced tumors (see below) is also localized on chromosome 2. The Gga 2 p3-2 zone represents a hot spot for MAV integration. Three BACs (85, 50 65) were mapped in this area. Bac 85 is a little more distal than the 3 others. BACs 50 and 65 are very close to each other but distinct. It is worth noting that Nal 2–28 (Pajer et al. in press) which include the twist gene overlaps with BACs 50 and 65. Our investigations also pointed out WTLS (at 7p11p15 in human) as a locus of interest for BACs 50 or 65.

Within the cytogenetic region Gga1q1 several genes of interest were potentially detected by FISH with BAC 22 and BAC 100. Among them, the cyclin-dependent kinase inhibitor 1B (CDKN 1B, 12p13 in human) and the N-ras oncogene (1p13 in human). In the same area, the data obtained by Pajer et al (in press) pointed to the POU2, OTF1, Oct1 transcription factors (1q22-3 in human). The localisation of BAC83 at Gga 1qter suggests as a potential target BIRC3 (Hsa 11q21) a candidate oncogene which is highly expressed in normal kidney and was reported to inhibit apoptosis.

At Gga3q24, the Wilms tumour 1 associated protein (WTAP, 6q25-27 in human) is a potential gene of interest for the region that is detected by two independant probes on BAC 71.

Two MAV1 insertion sites maped at 5q23-25 on the chicken genome. The human syntenic fragment (14q21-33), GPH (gephyrin at 14q23.3), TRAF3 (TNF receptor associated factor 3, at 14q32-33) and TGFβ3 (Transforming growth factor 3, at 14q24).

BACs 1 and 90 which maped at Gga 5 q23-25, contain MAV integration sites that were identified in two tumors representing different developmental stages. Both mapped very close to each other but did not co-localized since Bac1 is more proximal than Bac 90. The human syntenic fragment (14q21-33) contains several genes involved in kidney development and tumorigenesis. Among them, RCC2 (renal cell carcinoma 2, at 14q22-ter) is a locus which is lost in sporadic, non papillary renal cell carcinomas and oncocitomas. GPH (Gephyrin at 14q23.3) is a cytoplasmic, peripheral protein that anchors Gly-R. Although it is widely expressed, it is especially predominantly expressed in kidney. TRAF3 (TNF receptor associated factor 3 at 14q32-33) encodes an adapter protein that recruits other signaling molecules to the ligand-bound TNF family receptor. A gradient of TRAF3 is detected along the nephron, with progressive expression from proximal tubule to the collecting duct. TGFB3 is a well know transforming growth factor.

The region defined by BAC1 and 90 also corresponded to Nal 5–13 (Pajer et al. In press). Because these integration sites were identified in tumors representing different developmental stages, this area corresponded to a common integration region whose alteration is conserved during tumor progression, therefore suggesting that the gene(s) encoded by this portion of genome might be critical for nephroblastoma development and (or) tumor progression.

In addition to this situation, the two other integration sites identified in the most developed tumor by BACs 15 and 2 corresponded respectively to ccn3/nov (8q24.1 in human) and AdamTS1 (21q23.1 in human), two genes whose involvement in angiogenesis, matrix remodeling and tumorigenesis is well documented [7,19-27]. The ccn3 gene was previously mapped on chicken chromosome 2q34-36 [28]. Although the present study, and the results of Pajer et al. indicated that ccn3 is not a common integration site for MAV, this gene was identified as a MAV target in both studies. However, the MAV2-induced tumors analyzed by Pajer et al. did not show any increase in ccn3 expression.

Since both the MAV1- and the MAV2-induced nephroblastomas that we analyzed showed elevated levels of ccn3 expression [1], these conflicting observations result from either the route of injection, the time frame for injection, the different nature of the viral strains or host differences. The MAV2 (O) strain that was used in our previous studies was molecularly cloned and sequenced [2]. It induced 20% nephroblastomas, as opposed to 100% efficiciency of the MAV1(N) strain. In both cases, nephroblastomas were induced after intraveinous injection of 14 day-old embryos or intraperitoneal injection of day old chicken [1]. Since we have established that blastemal cells undergoing epithelial differentiation are the targets for MAV1, the time frame and route of injection may be critical. Indeed, the blastemal cells express high levels of ccn3 (Cherel et al. manuscript in preparation). Therefore, the elevated levels of ccn3 expression detected in all MAV-induced nephroblastomas might result from the expansion of blastemal cells that are transformed at a well defined stage of differentiation upon MAV1 infection.

Hybridization of BACs containing MAV1-integration sites with labeled mRNAs isolated from normal kidney tissue and nephroblastomas also permitted us to perform an analysis of the genes that are proximal to MAV integration sites and that are differentially expressed in normal and tumor condition. Among the different genes that were uncovered in this study, the ADAMTS1 gene was of potential interest. The ADAMTS1 protein is a matricellular proteinase known to participate in the late stages of tumorigenesis. Forty-five percent of newborn ADAMTS1 null mice died, probably as a result of kidney malformation that becomes apparent at birth [34].

Comparison of the expression pattern of CCN3 and ADAMTS1 shows striking similarities. In both cases overexpression of the protein is detected in all tumors tested, while the MAV proviral sequences are detected only once in the vicinity of these genes. These observations suggest that MAV -induced nephroblastoma occurs via a multistep process that involves a cascade of proteins acting along a common signaling pathway. Direct or indirect alteration of any step could result from MAV integration within or in the vicinity of critical genes whose increased expression would eventually be required for tumor progression. The identification of TGFβ 3 locus as a target for MAV integration in two independent tumors (501 and 725) is in favor of such an hypothesis. The role of TGFβ 1 in expression of CCN genes expression has been widely documented and the antagonistic activity of TGFβ 1 and TGFβ 3 has been shown to be critical in several instances. The activation of TGFβ3 expression by MAV might therefore result in an increased expression of CCN3 in tumors, similar to that observed upon integration of MAV within the ccn3 gene itself. Interestingly, tumor 725 which is the most developed is the only one in which integration of MAV occured in three gene loci whose alterations would have cummulative effects. A less developed tumor such as 501 only shows integration in the vicinity of TGFβ 3 and the early developed tumor does not show any of them.

In summary, our present study suggests that the development of nephroblastoma from an initial diffuse tumor phenotype (501D) to a well developed compact tumor (725) is accompanied by the selection of MAV integration sites in chromosome loci where genes involved in kidney differentiation are localized. The alteration of any of these genes by MAV integration at early stages of blastemal cell differentiation, would trigger the tumorigenic process. The multiplicity of potential genetic and cellular targets would provide support to the very high efficiency of MAV1 (N) which can induce 100% nephroblastomas within a 8-week period of time post injection. It will be interesting to determine whether the phenotypic variability of the MAV-induced nephroblastomas compares to the Wilms' tumors situation, and if the various subtypes of tumors result from different sequences of genes alterations.

## Competing interests

The authors declare that they have no competing interests.

## Authors' contributions

CLL carried out the isolation, characterisation of the BACs and performed the molecular studies; PC carried out fluorescent in situ hybridization experiments and participated to the editing of the manuscript;

AB provided funding and laboratory space to PC

VJ participated to the isolation of the cellular fragments that were further used as probes for BAC screening

CA hosted CCL for the BAC screening and participated to the initial design of the BAC studies

RZ was directly in charge of CCL training for the BAC library screening and coordinated the characterization of the BACs and designed the differential expression analysis

BP conceived the study design, coordinated and edited the manuscript, and obtained major sources of funding for this project.

## Supplementary Material

Additional File 1 Compilation of cytogenetic data obtained from FISH. For each chicken chromosome (Gga) the table shows the positions on which MAV sequences have been maped. From left to right : number of the MAV sequence in the BAC, origin of the tumor (501D: diffuse, 501 : intermediate, 725 well developed), number of the probe used to isolate the BAC, and cytogenetic information. For comparison, the information regarding the position of the Nals (Pajer et al. In press) has been also indicated. The positions of the Nals were calculated from their physical localization on the corresponding chromosome (for example, Nal 1–17 is on Gga 1 at 17 M p band, the Gga1 is 570 M bp long) and the corresponding band estimated by using the consensus mid point according to Schmid et al (2000). Human syntenic segments were also noted according to Schmid et al (2000). The identification of potential genes noted in italic was based on synteny and genome comparison. Click here for file
